# Puerperal Fever

**Published:** 1873-02

**Authors:** W. W. Vinnedge

**Affiliations:** Formerly Resident Physician in the Cincinnati Hospital


### PUERPERAL FEVER.

BY W. W. VINNEDGE, M.D.,

*Formerly Resident Physician in the Cincinnati Hospital.*

The question of the presence of contagion in puerperal fever is old, as well as important. Opinions of leading medical men are at variance on this subject, and not less so now than formerly. Anything, then, in the way of experience and observation will, I believe, be acceptable, the attention of your readers having recently been called to the subject by the excellent general article in the last number of the *Practitioner.* I believe experience in the Cincinnati Hospital through two separate epidemic attacks supports the doctrine of contagion, and it is to the course of the disease during these attacks that I desire to call attention.

Puerperal fever first appeared in the hospital during the month of January, 1869. At that time the building had been occupied but a few months, and even then only a portion of it was completed—the south pavilions. At the outbreak of the disease, the lying-in room occupied the third floor, Central avenue side, and was immediately above the male medical ward on the second floor, and the male surgical ward on the first floor. During the time the disease prevailed, several cases were reported—I do not now remember how many—and two deaths occurred. Soon after the disease manifested itself, it was discovered that the ventilation, which was new and had not been thoroughly tested, was faulty, and that foul air from the medical and surgical wards, to a greater or less extent, passed into the obstetric ward. As soon as this was ascertained, it was remedied, the patients moved to another portion of the house, and the disease ceased to exist. I may also say that at the time several cases of erysipelas, one of pyæmia, one of gangrene, and one of pyæmia following an operation for stone, existed in the surgical ward below. This was the explanation of the origin of the outbreak.

The disease appeared a second time in the obstetric ward, on the last day of October, 1869, attacking a primipara with chill four days after delivery. During the time of her attack—one month—seven well-defined cases were treated. No deaths occurred.

At this time the obstetric ward occupied the second floor, and was immediately above the female medical ward. As the disease manifested itself, it was ascertained that a severe case of erysipelas had been treated in the ward last named. It was also known that the ventilation in the female medical ward was defective, and I remember that it was charged that it was the only ward in the house where ventilation failed to approach satisfaction. Whatever the origin of the disease, once it was established, it attacked every woman delivered, and passed into the lying-in ward, notwithstanding the efforts made to prevent it by window ventilation, the liberal use of disinfectants, and perfect cleanliness. Finally, the women not already confined were removed to another part of the house, into an unoccupied pavilion, the puerperal patients being left where they were taken ill. Separate house physicians and nurses were assigned to the wards, and the disease disappeared.

Since I have referred to the influence of surgical air upon lying-in patients, I may give another instance having a bearing in the same direction—a slight one, it is true, but still noticeable. Late in the year, during the service of Dr. M. B. Wright, it was noticed that patients in the lying-in pay ward recovered from their confinements slowly, and that red tongue and frequent pulse were not uncommon. The explanation, it seemed to me, was to be found in the female surgical ward being on the floor immediately below.

The wards in this hospital do not communicate with each other except by winding stairway at one end, shut off by doors, one above and one below. At the time the disease prevailed it was asked, Did the obstetric assistant have anything to do in the spread of the disease by assisting in post-mortem examinations? To this I say no. A rule of the house positively prevented this; and I do not remember an instance where an assistant in the department of diseases of women and children assisted in an autopsy. Further, it was customary for the physician in attendance during the prevalence of the fever, or even of unpleasant symptoms, to wash his hands in a weak solution of carbolic acid before going from one patient to another.

I will not more than allude to the apparent connection between erysipelas, hospital gangrene, pyæmia, and other low forms of fever, and the disease under consideration. From a clinical lecture, delivered by Dr. Geo. Mendenhall, on puerperal fever, I learn that his experience in the Cincinnati Hospital with the affection was similar to that in the Florence Nightingale lying-in ward, which occupied the third floor of King's College Hospital, and which had medical and surgical wards below the obstetric.

In a foot-note in Bedford's Principles and Practice of Obstetrics, I find the following deductions from the researches of a German investigator on this subject: "Any fluid matter, in a state of putrefaction, communicated by linen, by a sponge, by a catheter, by small particles of the placenta, or even by the atmosphere impregnated with the foul substances, may produce puerperal fever."

Dr. Mendenhall, in the lecture already quoted, says: "With these views of the causes of puerperal fever, and of its pathology, we can hardly avoid the view of contagiousness, at least under certain circumstances."

In the practical application of this question, we confess ourselves able to think of nothing better than to follow the advice of Bedford: "It is the duty of the medical man, when in attendance on a woman attacked with puerperal fever, no matter what his views may be as to the contagiousness of the disease, to use every precaution against translating the affection through his own person. In this precaution nothing will be lost, and much may be gained."—*American Practitioner.*

